# The clinical outcomes of total vs. half patellar tendon transposition combined with robot-assisted medial patellofemoral ligament reconstruction and extensive lateral release for the treatment of habitual patellar dislocation in adolescents

**DOI:** 10.3389/fped.2025.1637470

**Published:** 2026-01-02

**Authors:** Qiuzhen Liang, Hongwei Zhan, Peidong Liu, Fujun Zhang, Zandong Zhao, Xin Kang, Chaofan Liao, Bin Tian, Jiang Zheng, Liang Zhang

**Affiliations:** 1 Sports Medicine Center, Honghui Hospital, Xi'an Jiaotong University, Xi'an, Shaanxi, China; 2 Department of Orthopedics, The Hospital of Xidian Group, Xi'an, Shaanxi, China

**Keywords:** habitual patellar dislocation, medial patellofemoral ligament, robot-assisted, total patellar tendon transposition, half patellar tendon transposition

## Abstract

**Purpose:**

This study reviews a series of adolescent patients with habitual patellar dislocation (HPD) who underwent combined surgical procedures and compares the clinical outcomes between a total patellar tendon transposition group and a half transposition group.

**Methods:**

A retrospective cohort study of skeletally immature patients with HPD who underwent either total patellar tendon transposition or half patellar tendon transposition, in addition to robot-assisted MPFL reconstruction and extensive lateral release, between May 2018 and May 2023. The vertical distance between Schoettle's point and the medial distal femoral physis was measured intraoperatively using a navigation system. Clinical outcomes were evaluated through imaging studies, physical examinations, and pre- and postoperative functional ratings.

**Results:**

A total of 58 patients were included in the final cohort. Among these, 30 patients were assigned to the total patellar tendon transposition group (TPTT group), while 28 patients were in the half patellar tendon transposition group (HPTT group). All patients returned for follow-up, at a mean of 31.5 ± 7.8 (range: 24–52) months after surgery. The average age of patients was 13.4 ± 1.7 (range: 11–16) years. The Schoettle's points were all located below the medial distal femoral physis, with a mean vertical distance of 5.88 ± 2.14 mm from Schoettle's points to the medial distal femoral physis. There was no recurrence of dislocation or severe complications; however, two patients (7.1%) in the HPTT group exhibited J sign. There was a statistically significant improvement in knee function scores among all patients (*P* < 0.001). At one-month postoperative assessment, the HPTT group demonstrated significantly higher Kujala scores compared to the TPTT group (*P* < 0.001). However, at final follow-up, the TPTT group showed superior functional outcomes, with higher Kujala (*P* = 0.033) and IKDC scores (*P* = 0.020) than the HPTT group. Compared with the preoperative results, there was a significant improvement in both the patellar tilt angle and the congruence angle (*P* < 0.001). No significant difference in the patellar tilt angle was observed between the two groups at the last follow-up (*P* = 0.730). Additionally, the congruence angle between the two groups showed a significant difference (*P* = 0.019) at the last follow-up.

**Conclusions:**

The combined procedures, which include extensive lateral release, total patellar tendon transfer, and robot-assisted MPFL reconstruction, can achieve good patellar tracking and stability in the treatment of HPD in adolescents, and may provide better clinical outcomes than the half patellar tendon transposition group.

## Introduction

Habitual patellar dislocation (HPD) in the adolescent population presents significant challenges due to its association with various abnormalities and an immature skeleton. Strong evidence supports early intervention for recurrent medial patellofemoral ligament (MPFL) dislocation in children, demonstrating high success rates and improved functional outcomes (1). Despite careful surgical planning and the use of physeal-sparing techniques to mitigate risks of growth plate disruption and graft failure, challenges such as avoiding overconstraint, the potential need for subsequent procedures, and prolonged rehabilitation periods persist (2). Some surgeons recommend postponing adolescent HPD surgery until the patient is older. However, frequent dislocations often interfere with daily activities, and patellofemoral joint instability may worsen with age. Therefore, early surgical intervention is advised, but it must be performed with caution. Combined surgical techniques are often necessary, as a single procedure is frequently insufficient to correct habitual dislocation (3). Combined procedures, such as tibial tubercle transfer and medial femoral patellofemoral ligament (MFPL) reconstruction using a femoral bone tunnel, are commonly utilized to treat adult HPD. However, in children and adolescents, these techniques may pose a risk to open growth plates.

Typically, at the femoral end, surgeons may employ techniques such as adductor magnus suspension, proximal tuber realignment, medial patella retinaculum reinforcement, and semitendinosus utilization to avoid the epiphyseal region (4); however, these techniques do not achieve anatomic reconstruction. The femoral insertion of the medial patellofemoral ligament (MPFL) is typically located within a well-defined 5-mm area, commonly referred to as Schoettle's point in contemporary literature (5). However, due to the intricate anatomy of the distal femoral physis, no definitive consensus has been established regarding the precise spatial relationship between the MPFL insertion and the physeal plate (6). Therefore, the robot-assisted reconstruction approach might be the most precise and secure. Based on the precise placement of the Schoettle point during the procedure, this technique can create customized bone tunnels to avoid damaging the epiphyses.

At the tibial end, transposing the half-patellar tendon [Roux-Goldthwait technique (7)] may create an effect similar to internal transposition of the patellar ligament, but the degree of transposition appears challenging to compare with that of tibial tubercle transfer in adults. Therefore, the combined procedures reported in this study, which include robot-assisted all-epiphyseal MPFL anatomic reconstruction and release of the lateral structures, as well as total patellar tendon transposition (TPTT) instead of tibial tubercle transfer, are noteworthy. To the best of our knowledge, TPTT or this combined surgery has not been reported in the literature to date. We hypothesized that robot-assisted MPFL reconstruction combined with total patellar tendon transposition would provide better clinical outcomes than the half patellar tendon transposition group.

## Patients and methods

**Figure 1:**
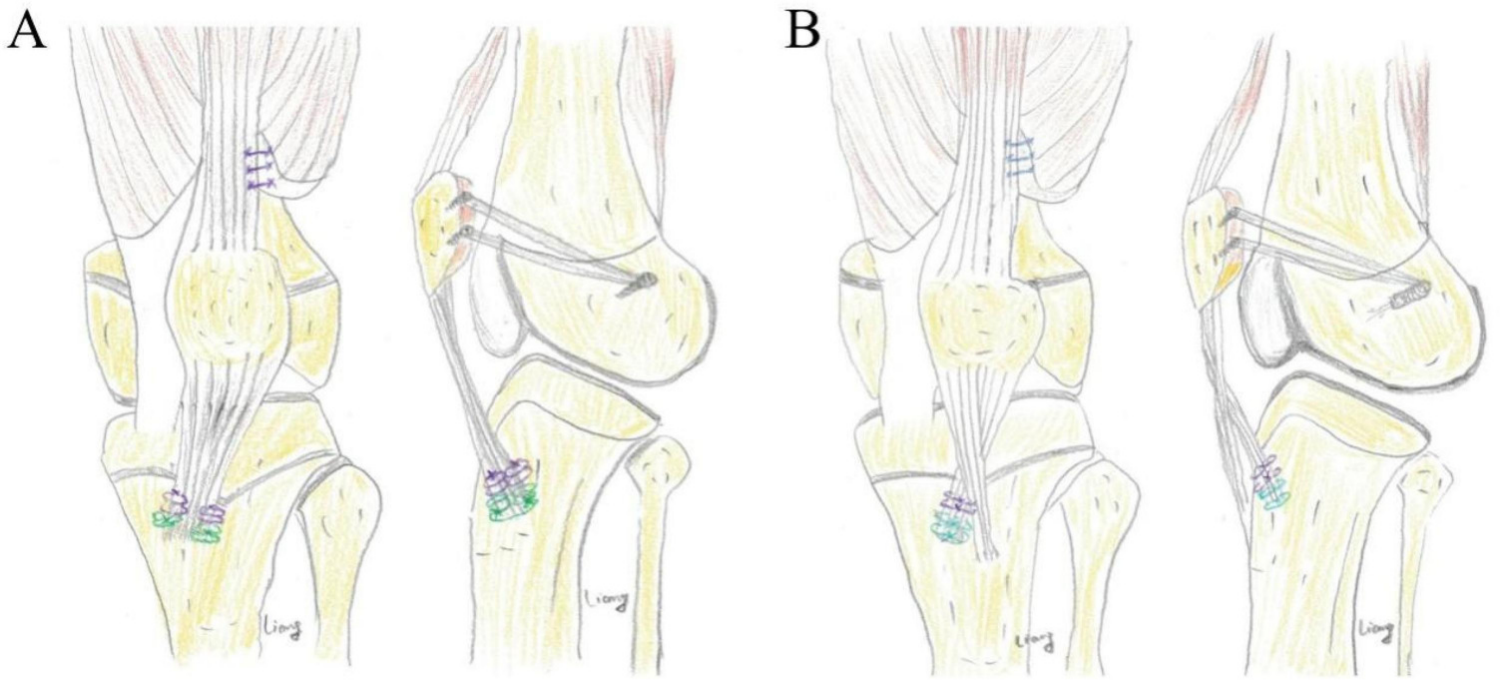
Schematic diagram of TPTT **(A)** and HPTT group **(B)** TPTT, total patellar tendon transposition; HPTT, half patellar tendon transposition.

This is a retrospective cohort study of skeletally immature patients with HPD who underwent either total or half patellar tendon transposition, along with robot-assisted MPFL reconstruction and extensive lateral release. The procedures were performed between May 2018 and May 2023 at a single institution by a single surgeon group. The study protocol received approval from the Ethics Committee. The initial records search identified 81 patients, and the inclusion criteria were as follows: (1) patients must be skeletally immature; (2) HPD occurs in both flexion and extension of the knee; (3) an autogenous hamstring tendon must be used. The exclusion criteria included: previous knee surgery (*n* = 7); revision surgery (*n* = 3), and Insall-Salvati ratio greater than 1.2 or less than 0.8 (*n* = 13). Patients whose patellar tendons were completely transposed constituted the total patellar tendon transposition (TPTT) group, while those whose patellar tendons were partially transposed were designated as the half patellar tendon transposition (HPTT) group (Figure 1). Pre-operative images were examined to assess growth plate status, lower extremity alignment, tibial tubercle-trochlear groove distance (TT-TG), Q-angle, angle of knee flexion during patellar dislocation, and anterior inclination of the femoral neck. Preoperative evaluations comprised clinical and imaging examinations, in addition to the Kujala score and the International Knee Documentation Committee (IKDC) score.

### TPTT group

Step 1: Extensive lateral release

A longitudinal skin incision approximately 15 cm in length was made from the lateral side of the proximal patella to the lateral side of the tibial tubercle. After releasing the contracture tissue between the iliotibial band and patella, the patellar attachment of the vastus lateralis muscle was cut in an L-shaped manner. Subsequently, a longitudinal release along the lateral margin of the rectus femoris tendon was performed, followed by a horizontal release to partially resect the distal end of the vastus lateralis tendon from the patellar side. Finally, a tension-free suture was performed at 90° knee flexion between the distal end of the vastus lateralis tendon and the proximal end of the rectus femoris.

Step 2: Total patellar tendon transposition

After the medial periosteum of the tibial tubercle was excised with a small blade, the patellar ligament was completely detached from the tibial tubercle. Four suture anchors were inserted into the medial aspect of the tibial tubercle, forming a rectangle approximately 1 cm by 2 cm on the medial side. This area served as the fixation site for the transposed patellar ligament following its medial and proximal displacement, which typically involved an internal displacement distance of about 1 cm. The proximal shift was minimal, usually not exceeding 0.7 cm. The distal, medial, and lateral edges of the transposed patellar ligament were securely anchored with sutures, and the periosteum was then sutured over the surface of the transposed patellar ligament using 3-0 sutures. After the above procedures, the surgeon pushes the patella with the thumb, and the patella can usually be in the range of full degrees without dislocation. If there is still significant dislocation of the patella at full knee flexion, partial central quadriceps extension may be considered.

Step 3: Robot-assisted femoral tunnel planning

**Figure 2:**
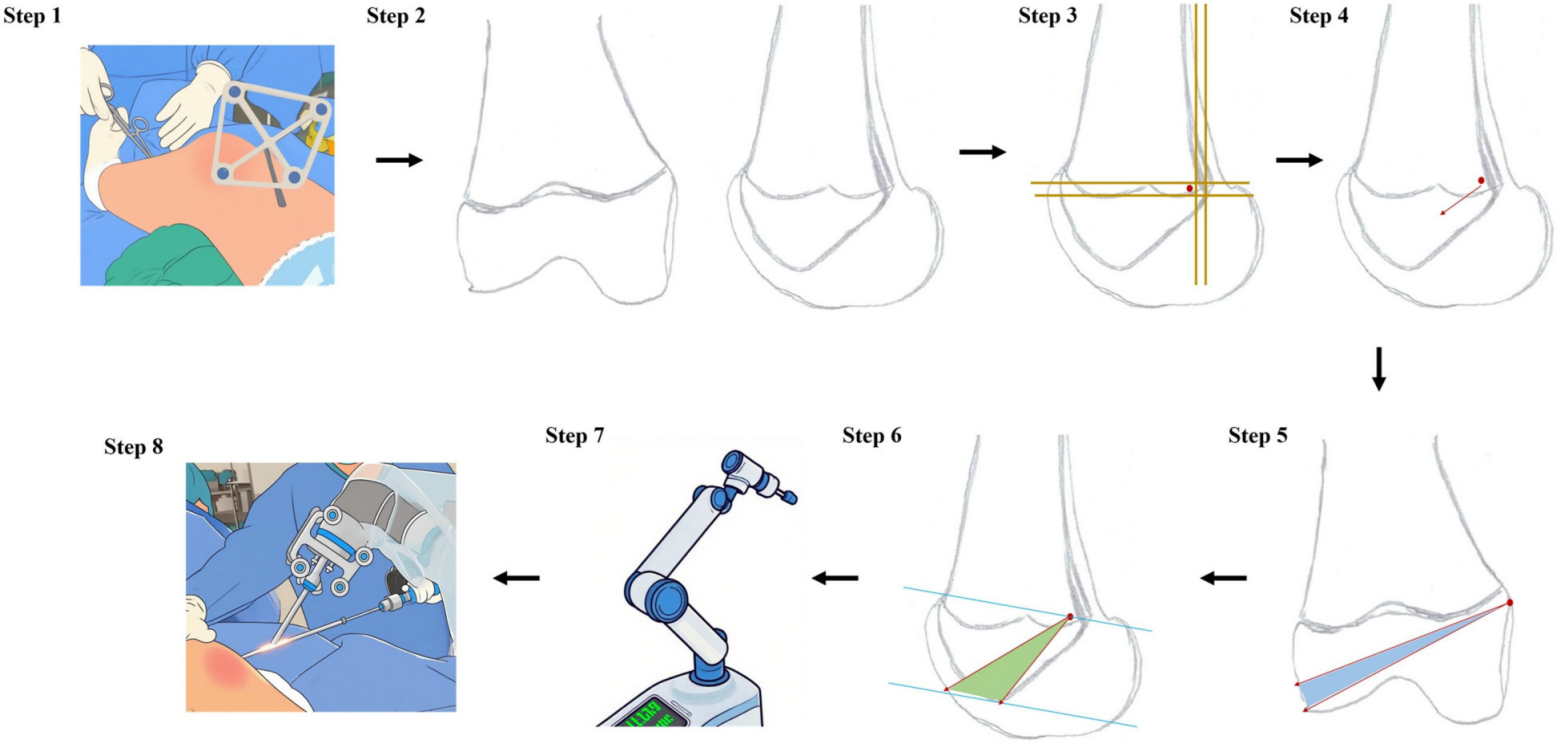
Diagram of the robot procedure.

The affected limb is elevated at the popliteal fossa to facilitate fluoroscopy, and after securing the ankle, the fluoroscopy machine, robotic arm, optical tracking device, and computer operating console are positioned. Specific operational steps are illustrated in Figure 2.

Step 1: After fixing the lower limbs, install the locator at the distal end of the femur; Step 2: During the operation, obtain standard anteroposterior and lateral views of the femur and input the images into the intraoperative navigation system (TINAVI Medical Technologies Co., Ltd); Step 3: Determine the Schoettle point within the intraoperative navigation system; Step 4: Plan the initial bone path from the Schoettle point toward the distal epiphyseal area of the epiphysis; Step 5: Precisely adjust the direction of the bone path into the correct position (the blue area indicates the adjustable range), ensuring that the growth plate, the intercondylar fossa, and the lateral femoral condyle joint surface are avoided; Step 6: Based on the bone path determined in Step 5, make adjustments on the lateral radiograph at this point to ensure the integrity of the condylar fossa and the joint surface (the green area indicates the adjustable range); Step 7: Send the path plan to the navigation system to trigger the mechanical arm to automatically adjust to the target angle; Step 8: Using the robotic arm for assistance, the surgeon precisely implanted the guide pin.

Step 4: MPFL reconstruction

**Figure 3:**
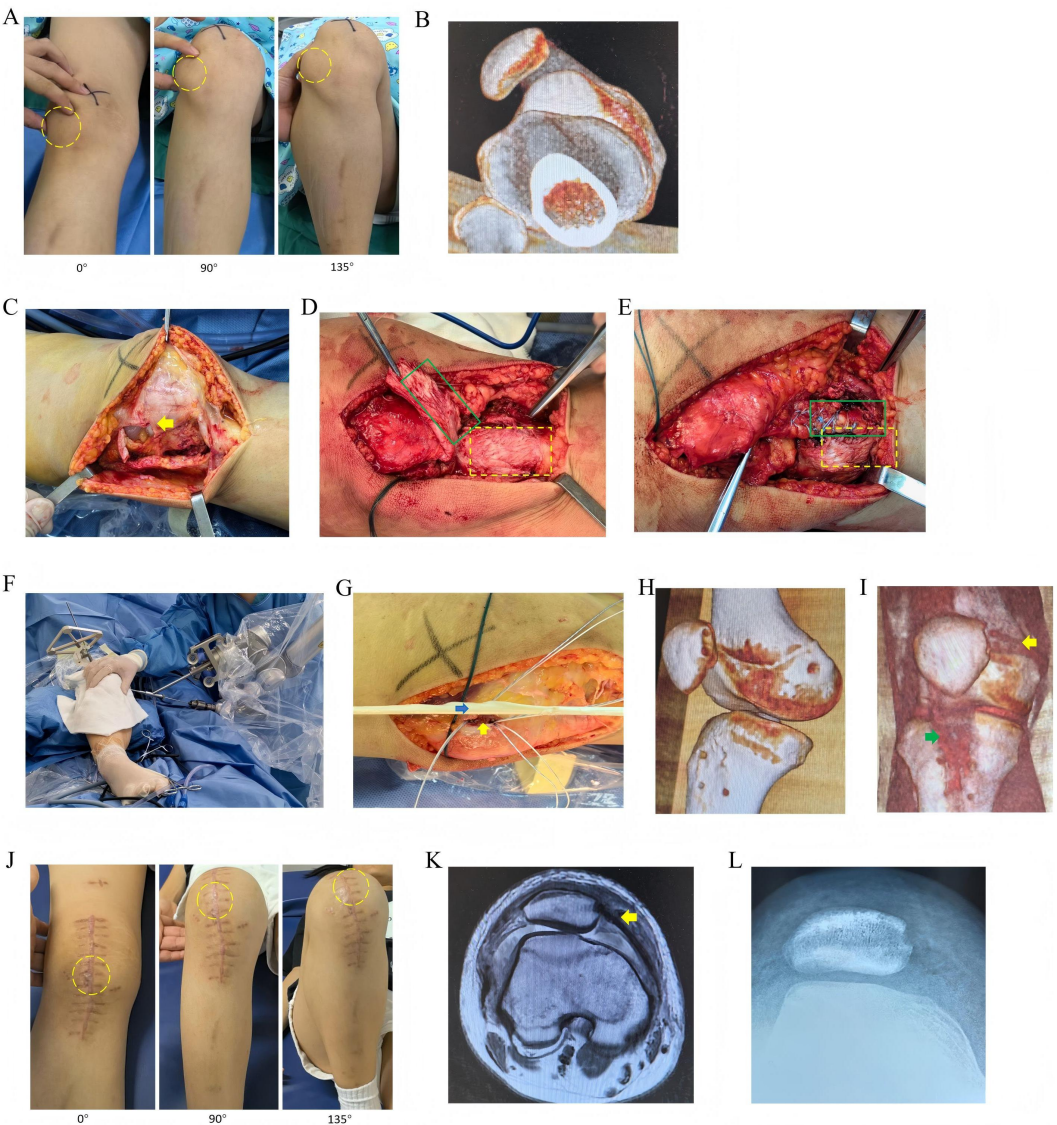
A 13-year-old female patient with habitual patellar dislocation underwent total patellar tendon transposition combined with robot-assisted medial patellofemoral ligament (MPFL) reconstruction. **(A)** Preoperative gross photographs revealed patellar dislocation at all angles of knee flexion and extension; **(B)** preoperative three-dimensional reconstructed CT images; **(C)** the extensive lateral release involved an L-shaped incision of the vastus lateralis muscle; **(D)** the entire patellar ligament (green rectangle) was detached from the tibial tuberosity (yellow rectangle); **(E)** the patellar ligaments were displaced medially and superiorly towards the tibial tuberosity (green rectangle) and secured with four anchors; **(F)** the MPFL femoral bone tunnel was prepared using the assistance of a robotic arm; **(G)** the bone groove (yellow arrow) along the medial margin of the patella was prepared, and the semitendinosus muscle (blue arrow) was secured using two anchors; **(H)** postoperative 3D reconstructed CT images demonstrated the femoral point of the MPFL, four anchor holes in the medial tibial tubercle, and two anchor holes along the medial margin of the patella; **(I)** post-operative 3D reconstructed CT images revealed both the MPFL (yellow arrow) and the transferred patellar ligament (green arrow); **(J)** two years after the surgery, the patellar trajectory was optimal, and there was no dislocation during flexion and extension; **(K)** MRI revealed the reconstructed MPFL (yellow arrow) two years after the surgery; **(L)** axial patellar radiographs showed a well-aligned patellofemoral joint two years after surgery.

After the guide pin is satisfactorily implanted, the medial tunnel is prepared using a 6 mm coarse drill, followed by the lateral tunnel prepared with a 2.5 mm fine drill, and a traction suture is introduced for later use. A 2 mm deep bone groove was created in the upper one-third of the superomedial aspect of the patella. Two anchors were individually placed to secure the middle portion of the semitendinosus tendon. After the isometric length of the reconstructed MPFL was determined through knee flexion and extension, the semitendinosus tendon was fixed at the Schoettle point using an interference screw, as shown in Figure 3.

### HPTT group

Step 3 of robot-assisted femoral tunnel planning, Step 1 of extensive lateral release, and Step 4 of MPFL reconstruction are identical to those in the TPTT group.

Step 2: Half patellar tendon transposition

**Figure 4:**
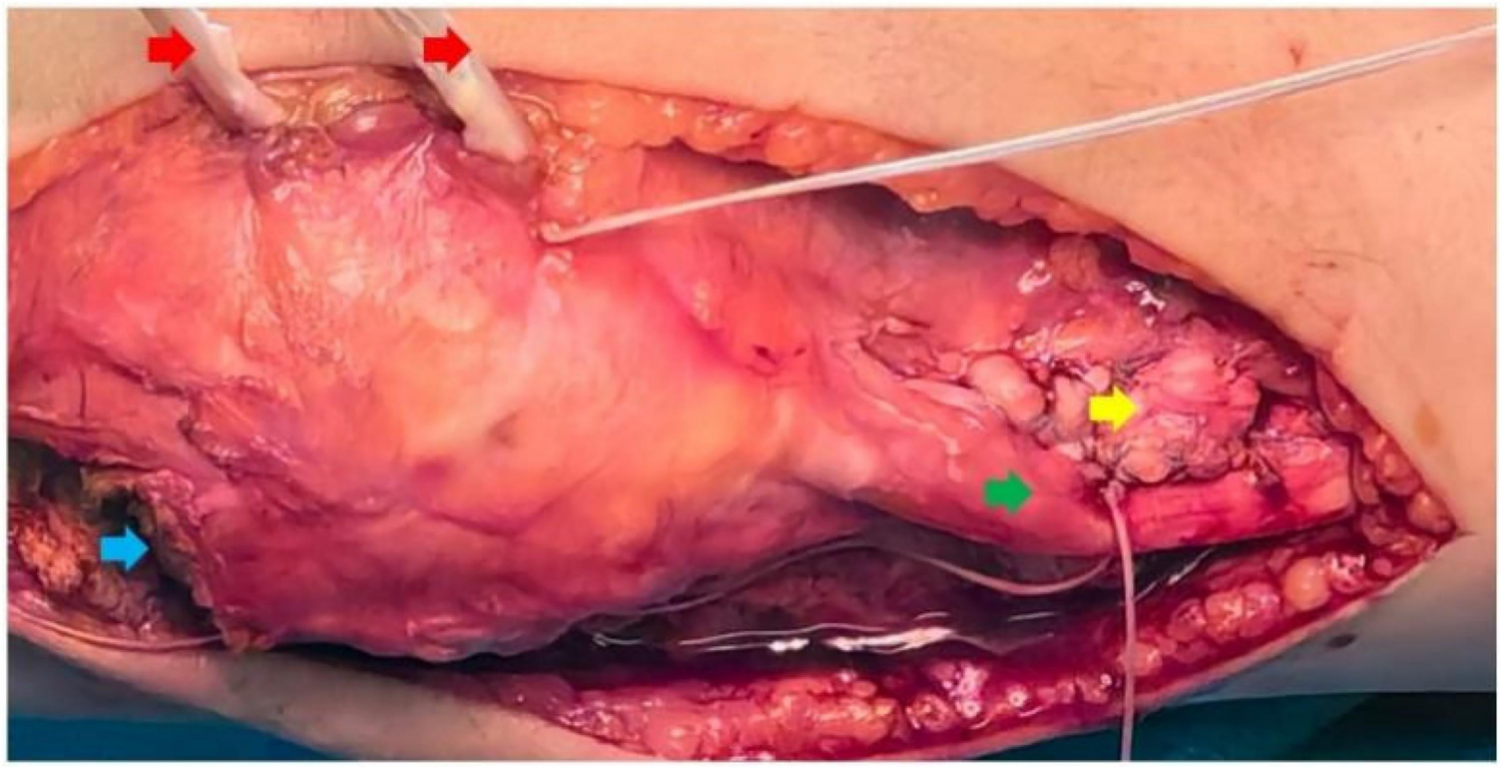
Half patellar tendon transposition (the red arrow indicates the MPFL; the blue arrow highlights the L-shaped incision of the vastus lateralis muscle; the green arrow points to the lateral half of the native patellar tendon; and the yellow arrow represents the half-transferred patellar tendon, which is sutured with two anchors).

Firstly, the lateral half of the tendon was longitudinally separated from the medial half and passed beneath the medial patellar tendon to the medial side of the tibial tubercle. After temporarily tensioning the transposed patellar tendon with tendon forceps on the medial side of the tibial tubercle, the periosteum on the medial side of the tibia was further detached. Under fluoroscopy, two suture anchors were inserted into the medial side of the tibial tubercle, with an approximate 1 cm difference in implantation distance between the two anchors. The braided tendon was then knotted, and finally, the periosteum was sutured to cover the surface of the patellar tendon. HPTT is illustrated in Figure 4.

### Post-operative rehabilitation

On the second day following surgery, patients were permitted to begin muscle-strengthening exercises. Partial weight-bearing with the support of a brace was also recommended at this time. Knee flexion should gradually increase, reaching the maximum angle within six weeks postoperatively. Subjective functions were evaluated using postoperative functional scores, including the IKDC score and the Kujala score. The congruence angle and lateral patellofemoral angle were measured using a CT scan, while the assessment of patellar tendon transposition was conducted through MRI.

## Results

There were 30 patients in the TPTT group and 28 in the HPTT group. The average age of the patients was 13.4 years (range: 11.1–16.0 years). No statistical differences were observed in the baseline data between the two groups (Table 1). All patients returned for follow-up, at a mean of 31.5 ± 7.8 months (range: 24–52 months) after surgery. The Schoettle's points were all located below the medial distal femoral physis, with a mean vertical distance of 5.88 ± 2.14 mm (range: 3.21–9.83 mm) from Schoettle's points to the medial distal femoral physis. During follow-up, the IKDC score and Kujala score were significantly improved compared with the preoperative scores (*P* < 0.001) at the last follow-up. At one-month postoperative assessment, the HPTT group demonstrated significantly higher Kujala scores compared to the TPTT group (*P* < 0.001). However, at final follow-up, the TPTT group showed superior functional outcomes, with higher Kujala (*P* = 0.033) and IKDC scores (*P* = 0.020) than the HPTT group. All the patients have resumed full activities with satisfactory knee function. At the last follow-up, two patients (7.1%) in the HPTT group exhibited slight residual abnormalities (J sign) in the patellar tracking, while the rest of the patients showed normal patellar tracking. Compared with the preoperative results, there was a significant improvement in both the patellar tilt angle and the congruence angle (*P* < 0.001). However, no significant difference in the patellar tilt angle was observed between the two groups at the last follow-up (*P* = 0.730). Additionally, the congruence angle between the two groups showed a significant difference (*P* = 0.019) at the last follow-up (Table 2). During follow-up, no patients developed patellofemoral arthritis, and there were no cases of recurrent dislocation or severe complications. One patient (3.6%) in the HPTT group developed fat liquefaction, which resolved after debridement.

**Table 1:** Patients demographics.

Group	Gender (Female/Male)	Age (year)	TT-TG (mm)	Anterior inclination of femoral neck (°)	Q-angle (°)	Dejour classification (B/C/D)	Kujala score	IKDC score	CA (°)	PTA (°)
TPTT	18/12	13.2 ± 1.9	23.6 ± 3.8	17.7 ± 5.6	12.1 ± 5.7	10/16/4	36.1 ± 10.9	40.5 ± 10.0	69.3° ± 15.2°	50.1° ± 13.2°
HPTT	17/11	13.6 ± 1.6	22.3 ± 3.5	15.9 ± 4.8	13.4 ± 4.8	10/14/4	39.2 ± 9.1	43.1 ± 11.7	66.4° ± 12.4°	46.6 ± 11.0°

TPTT, total patellar tendon transposition; HPTT, total patellar tendon transposition; IKDC, International Knee Documentation Committee; CA, congruence angle; PTA, patellar tilt angle. Results are presented as *n* (%) or mean ± standard deviation unless otherwise indicated.

**Table 2:** Comparison of baseline data between the two groups.

Scoring/Parameters	TPTT group	HPTT group	*P* value
Kujala score (1 month)	44.1 ± 6.0*	49.8 ± 5.2	<0.001
Kujala score (last follow-up)	89.3 ± 7.8*	84.6 ± 8.8	0.033
IKDC score (1 month)	46.4 ± 6.8	48.7 ± 8.3	0.244
IKDC score (last follow-up)	91.6 ± 7.3*	87.0 ± 7.5	0.020
CA (last follow-up)	−1.7 ± 4.1*	0.75 ± 3.8	0.019
PTA (°) (last follow-up)	12.5 ± 7.2	13.1 ± 5.9	0.730

TPTT, total patellar tendon transposition; HPTT, total patellar tendon transposition; IKDC, International Knee Documentation Committee; CA, congruence angle; PTA, patellar tilt angle; Results are presented as *n* (%) or mean ± standard deviation unless otherwise indicated.

* *P* < 0.05, compared with HPTT group.

## Discussion

The most important finding of this study was that the combined procedures including extensive lateral release, total patellar tendon transfer, and MPFL reconstruction can achieve good patellar tracking and stability in the treatment of HPD in children and adolescents. Moreover, total patellar tendon transfer provides better clinical outcomes than partial patellar tendon transfer.

Since no single therapy is universally effective for adult HPD, a combination of surgical procedures is typically recommended. However, despite the numerous techniques described in the literature, managing HPD in skeletally immature patients remains considerably more challenging than in adults (8). Similar to adults, for skeletally immature patients, extensive release of the contracted lateral structures and extension of the knee extensors are of primary importance, as the main pathology of HPD involves tight lateral patellar retinaculum, fascia bands, and vastus lateralis (9). In the present study, all cases underwent varying degrees of tendon elongation, including patellar tendon transposition. However, central quadriceps extension was not necessarily required. In most cases, extensive lateral release involves an L-shaped cut in the vastus lateralis muscle, combined with either half or total patellar tendon transposition, allowing the patella to maintain its position typically within the range of 0–120° without dislocation. Following transposition of the patellar tendon, patellar tracking typically improves significantly. During intraoperative assessment, the surgeon applies medial pressure to the patella with the thumb along its lateral edge. Normally, the patella should glide freely without subluxation or dislocation. Persistent dislocation suggests insufficient lateral release. More importantly, the femoral screws for MPFL attachment were finally fixed at a knee flexion angle of 30° after cycling the joint through several flexion-extension cycles. This technique ensures moderate graft tension by avoiding fixation under forceful pulling.

Among various techniques, tibial tubercle transfer can alter patellar trajectories and extend the knee extensor mechanism by moving the tuberosity upward and inward. However, successful surgical techniques used in adults such as tibial tubercle transfer may endanger the open growth plate when used in adolescent, therefore, soft tissue procedures are more likely to be appropriate when significant growth remains (10, 11). Some authors considered that tendon lengthening is an essential part of the procedure to achieve proper patella tracking (12). The technique reported in this study not only extends the knee extensor mechanism but also directly shifts the patella inward. However, TPTT patients may experience early knee extension weakness, initially leading to lower functional scores compared to HPTT patients. Nonetheless, functional exercise can gradually return to normal over time.

In the present study, both total and half patellar tendon transposition techniques were employed. However, despite the good patellar tracking and stability achieved during the operation in both groups, the TPTT group exhibited higher functional scores and no instances of poor patellar tracking at the last postoperative follow-up. The higher score may be attributed to reduced pressure on the patellofemoral joint at high flexion angles or a more favorable correlation in the patellofemoral relationship in TPTT group. The maltracking (J sign) in HPTT group may be caused by the following reasons: 1) TPTT technique is comparable to extending the knee extension device, resulting in relatively small external and upward pull forces on the patella during active knee joint extension; 2) Transposing the full patellar tendon can more effectively shift the patella medially, maintain higher tension in the lateral vastus muscle extended by the L-type release, and ultimately improve the extension of the lateral side of the knee extensor device. In contrast, transposing only half of the patellar tendon may lead to subsequent contractions of the lateral vastus muscle released during the procedure, resulting in a J-sign.

The tendon-to-bone healing of the transferred tendon is another aspect that requires additional attention. Initially, we also had reservations regarding the healing of the patellar tendon. For this reason, we started by performing only half patellar tendon transfers. However, we later observed that in severe cases, half transfer was insufficient to restore proper patellar tracking. In half transfer cases, the transferred tendons healed well. This can be attributed to the secure suturing achieved using suture anchors, which fix the tendon firmly against the cortical bone. Additionally, the repositioned tendon is covered by the periosteum, thereby enhancing its blood supply. Together, these measures contribute to improved healing. For a half patellar tendon transfer, two anchors were used, while a total transfer requires four anchors fixed in a rectangular configuration (at each corner). The suture is then passed toward the center of the patellar tendon and returned to the four corners for suturing, followed by tying and fixation. This results in a relatively stable construct. Additionally, postoperative bracing provides protective support. Crucially, robust healing of the transferred tendon was observed in all cases. This highly promising result should provide surgeons with the confidence to attempt this technique.

In addition to extensive release of the contracted lateral structures and extension of the knee extensors, MPFL reconstruction remains the most crucial surgical procedure. The MPFL is the primary medial static stabilizer of the knee, making its reconstruction the most common and successful isolated surgical procedure for patellar instability (13). MPFL reconstruction techniques, which are effective in adults, carry the risk of growth plate injury when applied to skeletally immature patients. Moreover, the outcomes of these procedures in this population have not been thoroughly studied. It was previously believed that soft tissue surgeries, such as primary repair, medial retinacular imbrication, adductor magnus suspension, cerclage suture fixation around the medial collateral ligament, and other methods utilizing soft tissue attachment to the femur, would be a more appropriate approach to minimize the risk of femoral physis injury (13–16). However, these techniques depend on soft tissue sutures, which may be less reliable than bone tunnel fixation. Furthermore, anatomical restoration of the MPFL can be difficult to achieve with soft tissue techniques. Given the high failure rate of these soft tissue operations, anatomical repair is recommended for skeletally immature patients (6).

The femoral insertion of the MPFL is most commonly identified within a 5 mm region referred to as Schoettle's Point (5). Nevertheless, there is no consensus regarding its spatial relationship to the distal femoral physis (1). In the current study, a real-time computer navigation system was used to create a patient-specific bone tunnel for Schoettle point. This technique ensured that the lateral femoral condylar cartilage surface, intercondylar notch, and epiphysis remained unaffected. Our results, which indicate that the MPFL origin footprint is situated below the physis, are consistent with the majority of the literature (17). Furthermore, this study demonstrated through an intraoperative robotic navigation system that Schoettle's point can be safely targeted even in patients with open physes. To the best of our knowledge, total patellar tendon transposition has not been reported in the literature. When combined with robot-assisted MPFL reconstruction, it can achieve good patellar tracking and stability in the treatment of habitual patellar dislocation in adolescents.

In our previous report (18), robotic-assisted surgery was successfully used to treat pediatric recurrent patellar dislocation. Compared with current studies, although robotic surgery has been applied in both scenarios to avoid the physis and reconstruct the MPFL, we believe that robotic-assisted surgery holds greater value in the treatment of HPD in children. Unlike recurrent patellar dislocation cases, HPD is often accompanied by more severe femoral condyle deformities. However, existing robotic surgical systems fail to adequately visualize the specific deformed areas of the femoral condyle, articular surface, or trochlea. Conventional imaging may obscure the actual deformity due to factors such as overlapping femoral condyles, potentially leading to irritation of the articular surface or physis. To avoid such situations, during combined surgery, we schedule the robotic-assisted MPFL reconstruction as the final step (Steps 3 and 4), while performing extensive lateral release as the first step (Step 1). This allows for macroscopic observation to identify the developmental status and degree of deformity of the femoral condyle in advance, predict the insertion angle of the guide pin, and further assess whether the guide pin placement may cause damage to the intercondylar notch, articular surface, or physis. Additionally, in this study, some cases exhibited extremely poor development of the femoral condyle, making it impossible to apply the previously reported method for determining the optimal angle. However, by combining macroscopic observation, we were able to provide recommendations for distal and anterior tilt angles of the guide pin, followed by computer assistance to ensure surgical safety.

## Limitations

Due to the significantly lower incidence of this condition in children and adolescents compared to recurrent patellar dislocation, along with its more severe degree of dislocation, it is challenging to conduct randomized controlled studies at a single center. In the future, as technology advances and becomes more widely adopted, multicenter randomized controlled studies are expected to be carried out. Furthermore, long-term follow-up, will be necessary to assess whether patellar stability can be maintained without the onset of patellofemoral arthritis or other complications. Additionally, although robot-assisted surgery may initially be more time-consuming, its efficiency should improve with technological development. Prospective, randomized clinical studies would be warranted to further assess the procedure's clinical outcomes. Finally, in combined surgical procedures, it is difficult to clearly determine the specific contribution of the total patellar tendon transfer. This may require further biomechanical studies for verification.

## Conclusion

The combined procedures, which include extensive lateral release, total patellar tendon transfer, and MPFL reconstruction, can achieve good patellar tracking and stability in the treatment of HPD in children and adolescents. Additionally, in patients with open epiphyses, the robot-assisted technique for customized anatomic MPFL reconstruction, which employs a forward distal oblique bone tunnel, has demonstrated both safety and efficacy. We recommend this combined surgical approach for children and adolescents with HPD.

## Data Availability

The original contributions presented in the study are included in the article/Supplementary Material, further inquiries can be directed to the corresponding author.
